# High contrast breast cancer biomarker semi-quantification and immunohistochemistry imaging using upconverting nanoparticles

**DOI:** 10.1364/BOE.504939

**Published:** 2024-01-19

**Authors:** Sanathana Konugolu Venkata Sekar, Hui Ma, Katarzyna Komolibus, Gokhan Dumlupinar, Matthias J. Mickert, Krzysztof Krawczyk, Stefan Andersson-Engels

**Affiliations:** 1 Biophotonics@Tyndall, IPIC, Tyndall National Institute, Lee Maltings Complex, Dyke Parade, T12R5CP, Cork, Ireland; 2 Department of Physics, University College Cork, College Road, Cork, T12 K8AF, Ireland; 3 Lumito AB, Mårtenstorget 5, SE-223 51 Lund, Sweden

## Abstract

Breast cancer is the second leading cause of cancer death in women. Current clinical treatment stratification practices open up an avenue for significant improvements, potentially through advancements in immunohistochemistry (IHC) assessments of biopsies. We report a high contrast upconverting nanoparticles (UCNP) labeling to distinguish different levels of human epidermal growth factor receptor 2 (HER2) in HER2 control pellet arrays (CPAs) and HER2-positive breast cancer tissue. A simple Fourier transform algorithm trained on CPAs was sufficient to provide a semi-quantitative HER2 assessment tool for breast cancer tissues. The UCNP labeling had a signal-to-background ratio of 40 compared to the negative control.

## 1. Introduction

Accounting for 0.5 million deaths per year internationally, breast cancer is the most common form of cancer among women and constitutes a significant global health issue [1]. This high death toll persists even though the majority of women are now diagnosed with early-stage breast cancer, which is often curative with surgery, radiation therapy, and systemic therapy. Standard immunohistochemistry (IHC) labeling of tumor tissue biopsies, obtained by imaging molecular targets such as the estrogen receptor (ER), progesterone receptor (PR), and human epidermal growth factor receptor 2 (HER2) facilitates oncologists in determining both prognosis and treatment strategies for individual patients with breast cancer. The main advantages of the IHC technique lie in its high sensitivity and specificity, application to routinely available formalin fixed paraffin embedded samples, correlation to morphology and cost effectiveness [2]. However, despite its widespread use and incorporation into clinical practice guidelines, standard IHC has inherent limitations including low dynamic range, issues with quantification, subjectivity, multiplexing and co-localization [3–6].

For example, in a typical IHC workflow to determine the HER2 expression level a horseradish peroxidase 3,3’-diaminobenzidine (DAB) staining is used. The enzymatic oxidation of DAB generates an insoluble brownish precipitate at the location of the analyte. The completeness of the DAB precipitation along the cell membrane and the strength of the brown color are analyzed visually by pathologists to rather subjectively determine the HER2 level in a four category scoring system (0 to 3+) according to the current guidelines of American Society of Clinical Oncology/College of American Pathologists (ASCO/CAP) [7,8]. According to those guidelines, only HER2 3 + case, with complete and intense circumferential membrane staining for over 10% of the tumor cells, is considered HER2 positive. Currently, approximately 15% of women are diagnosed with HER2 positive breast cancer and receive HER2-targeted therapies such as trastuzumab, pertuzumab, or lapatinib [9]. The remaining 85% of diagnosed women fall into the negative category despite the fact that they can express weak to moderate staining in over 10% of tumor cells (cases of HER2 1 + and 2+). Since the detection of HER2 3 + cases has been of greatest clinical significance so far, the optimization of HER2 testing has always been focused on differentiating positive from negative cases rather than four distinct categories [10,11]. The recent introduction of new therapies, for example antibody conjugates like trastuzumab-deruxtecan, successfully targeting the sub-group of patients with lower HER2 levels [10,12–14], poses a new challenge of accurate and reproducible assessment of HER2 scoring at its lower end. The high rate of discordance among pathologist scoring lower HER2 levels (0, 1+, 2+) and poor interrater reliability suggest that traditional IHC doesn’t provide sufficient accuracy for clinical decision making [5,15].

The challenges associated with chromogenic staining, such as DAB, open up a new avenue for improved quantifiable labling schemes and intelligent image analysis techniques to enable extraction of previously hidden information. In the last decade vast efforts have been undertaken to utilize machine learning (ML) and artificial intelligence (AI) algorithms based on conventional histopathological staining to obtain more objective diagnostic results, including prediction of diagnosis, prognosis and treatment response [16–22]. At the same time, immunofluorescence (IF) using fluorophore-labeled antibodies has become an alternative approach. Nevertheless, organic dyes typically used in IF exhibit quenching and broad emission, which makes them not suitable for quantitative analysis and multiplexing [23]. Advances in nanoparticle functionalization and bioconjugation, however, has laid the foundation for a new generation of fluorescent nanoprobes [24]. Numerous groups have explored the possibility of using quantum dots (QDs) [25–28] and Raman probes [29,30] for improved profiling of multiple biomarkers, but ideal label providing high contrast and high dynamic range imaging has yet to be found.

Upconverting nanoparticles (UCNPs) have recently emerged as a versatile fluorescence imaging platform with unique photophysical properties desirable in biomarker detection and imaging applications [31–34]. Due to their anti-Stokes shift, the autofluorescence of the background can be avoided yielding very high sensitivity, even down to single particle detection [35,36]. This, in turn, enables high contrast measurements providing quantitative information about biomarker concentration and together with the narrow emission lines opens up the possibility of biomarker multiplexing. Moreover, the extremely high photostability of UCNPs is an advantage in comparison to other fluorescent dyes [37,38]. These features make UCNPs ideal candidates to explore the avenue of digital pathology with multiplexed, sensitive and highly specific molecular detection. To date, the application of UCNP conjugates with different breast cancer cell lines have demonstrated excellent results with highly specific detection of the HER2 biomarker, yielding a 50-fold improvement in signal-to-noise ratio as compared to conventional fluorescent labeling under the same experimental conditions [39]. In addition, a multiplexed detection scheme of three common biomarkers, ER, PR and HER2, has been successfully demonstrated by employing the UCNP platform on breast cancer cell lines [40]. However, the majority of UCNP-biomarker labeling studies presented thus far use cancer cell lines rather than more complex histological samples and do not address the issue of biomarker quantification.

This study demonstrates the feasibility of using UCNPs as a promising candidate for high contrast breast cancer tissue labeling and brings up the potential for quantification of HER2 expression as opposed to standard DAB labeling. The novelty of the present work lies in the detection and semi-quantification of four distinct levels of HER2 (0, 1+, 2+, 3+) expressions in HER2 control pellet arrays (CPA), and in the demonstration of HER2 mapping in breast cancer tissue slides. In addition, a Fourier-transform-based image analysis method was developed to address the challenge of quantification of various levels of HER2 expressions. The imaging contrast achieved in the UCNP-labeled breast cancer tissue is compared with DAB-labeled slides and the contrast gain of UCNP over DAB is quantified.

## 2. Experiment description

**Fig. 1.**
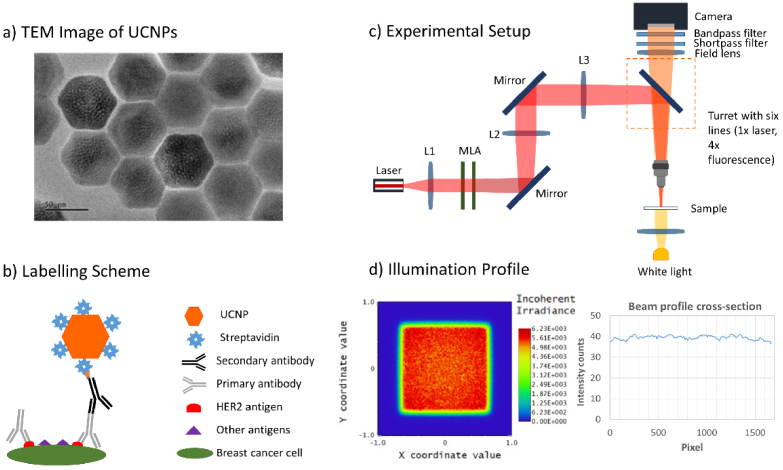
a) TEM images of UCNPs used in this study b) scheme of the HER2 labeling with UCNP-streptavidin conjugates c) multimodal laser illumination microscope system (Lens - L1, L2, L3, microlens array – MLA) d) uniform beam profile (Zemax simulation) and characterization of the beam profile of the 976 nm laser excitation line on the sample plane of the microscope.

We will here give a brief description of the study, with full details provided in the Supplement 1 S1. To investigate the suitability of UCNPs for the quantification of HER2 presence, CPAs (HistoCyte Laboratories Ltd, HCL028) with different levels of HER2 expression were incubated with anti-HER2 rabbit antibody, biotinylated anti-rabbit antibody, and finally labelled with streptavidin-PEG-UCNPs (NaYF4, with 2% Tm3 + and 18% Yb3 + doping). The average size (diameter inner circle) of UCNPs were around of 54** **nm ± 1.7** **nm (N = 155) as verified by TEM (Fig. 1(a)). The UCNP-streptavidin labeling scheme is depicted in Fig. 1(b). In the first step, a primary rabbit anti-HER2 antibody binds to the HER2 antigen localized in the cell membrane. A secondary anti-rabbit antibody modified with biotin acts as an anchor to the UCNP-streptavidin conjugates. The excess UCNP labels are removed in a washing step. In addition, samples were counterstained with DAPI. For demonstrating on real HER2 breast cancer tissue, HER2 3 + breast cancer tissue slides were used for this study and they were labelled using the same process as described above.

The slides are subsequently analyzed employing a microlens array (MLA) based Köhler-illumination type user-built microscope system. The schematic of the microscope system is shown in Fig. 1(c). The home-built microscope is connected diode laser source (Ostech dst11-150W-976nm-105µm), and beam shaped with set of lenses L1 (AC254-050-B-ML, f = 50 mm), L2 (LA1484-B-ML, f = 300 mm), L3 (LA1433-B-ML, f = 150 mm). The power and power density at the sample plane were approximately 10 W and 22.6 MW/m^2^, respectively. The emission from the sample is selected using shortpass filter at 900 nm and bandpass filter (bandwidth 40 nm, central wavelength 800 nm) and further imaged onto sCMOS camera (Photometric Prime BSI). The system and its optical components were optimized in the Zemax OpticsStudio software to provide a uniform beam profile. Figure 1(d) shows the results of simulations and experimentally measured uniform beam profile on a UCNP test sample. The microscope includes additional excitation lines to perform H&E, DAPI, and DAB imaging.

**Fig. 2.**
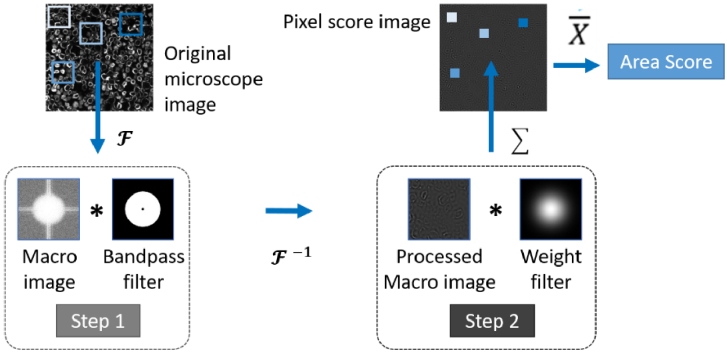
Flow chart of the Fourier-transform image processing algorithm for the semi-quantification of the HER2 expression.

To semi-quantify the UCNP labeling in the CPAs, the camera dark counts were first subtracted from recorded luminescence images. A schematic layout of the subsequent steps for semi-quantitatively assess the HER2 score in small sub-areas across the analysed images is shown in Fig. 2. First, a subset of macro images of 201 × 201 pixels with centre at each pixel to be analysed were generated. The size of the macro images was selected to include a number of neighbouring cells, minimize any distortion to the subsequent Fourier transform due to the finite size of the image and to also have one center pixel. In step 1, each macro image was 2D Fourier transformed and a band-pass filter was applied in the spatial frequency domain. This operation suppresses contributions of low frequency components, like background signals, while retaining the high-frequency components like the UCNP labeling of the cell membranes, and the completeness of UCNP HER2 labeling around the cell membrane. The parameters of the Fourier bandpass filter (inner and outer radius) were optimized to maximize the variation among the HER2 expression levels and capture the frequencies corresponding to circular HER2 expression around the cell membrane. Hence, the circular, high HER2 expression like 3 + has a relatively high contribution to filtered Fourier domain image as compared to non-circular lower HER2 expression (e.g. 1+, 0).

In step 2, the information is subsequently transformed back to the spatial domain and multiplied with a Gaussian weight filter with sigma value of 34 pixels. The weight filter ensures that the contribution of centre pixels in the macro image are dominating. All the 201 × 201 pixel values are then summed to create a single value for each macro image. This value is defined as the pixel score for the centre pixel, and the corresponding image generated by processing all macro images to pixel scores is called the pixel score image. Then area score is calculated as the mean of all pixels in pixel score image**.**

## 3. Results and discussion

**Fig. 3.**
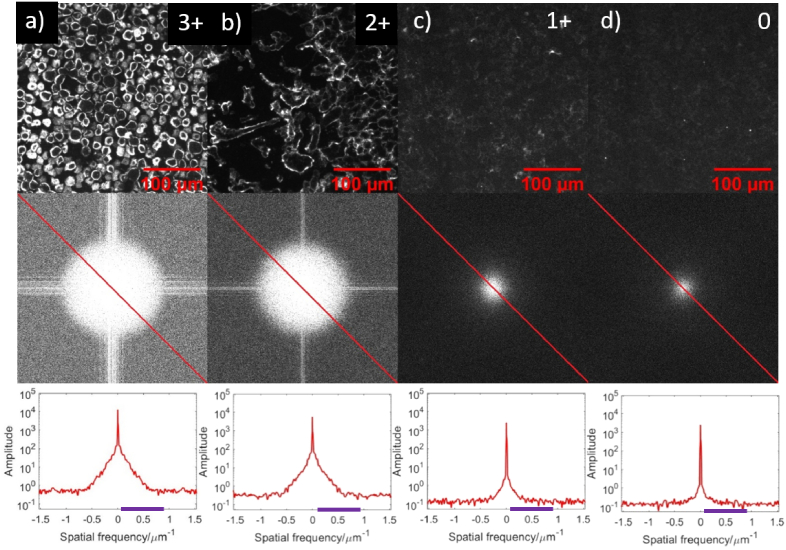
Microscope images, corresponding 2D Fourier transforms of the images, and cross sections line profile through these transforms (band pass filter range is highlighted with a violet bar at the axis) for UCNP labeled HER2 CPA samples with different levels of HER2 expression a) 3+, b) 2+, c) 1+, and d) 0. Note: Top row images (c, d) has different intensity scale as compared to a, b to improve visualization of the images

**Fig. 4.**
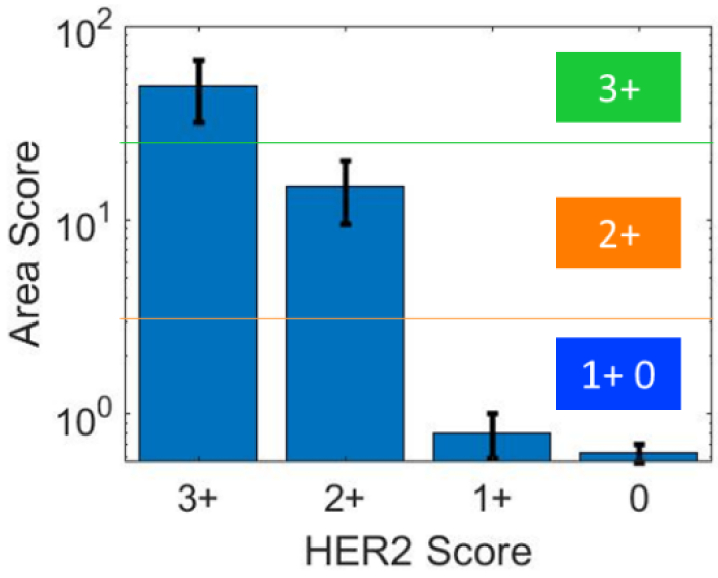
Results of the HER2 quantification for different HER2 expression levels in a CPA with standard deviation as error bars and thresholds for semi-quantification of HER2 expression.

For the bandpass filter in the spatial frequency domain of the images, an inner radius of 0.015** **µm^-1^ and an outer radius of 0.92** **µm^-1^. The lower limit was chosen to eliminate the zero DC frequency and the upper limit is chosen as the highest frequency seen in HER2 3 + expression. Figure 3 a-d shows the results of UCNP labeled CPAs with Fourier transformed image and line profile of frequencies. Here, the completeness of the cell membrane and intensity of labeling is decreased with declining HER2 level from 3 + to 2 + which decreases further in HER2 1+, 0 samples as expected by the ASCO/CAP guidelines [41]. In other words, fewer UCNPs are bound to cells in the HER2 control pellets exhibiting lower HER2 expressions. The area score with standard deviation across the pixel score images for different HER2 expressions in CPA samples is shown in Fig. 4. The area score provides a quantitative estimate of the HER2 expression for the entire CPA and increases with elevated HER2 expression levels. Thanks to the choice of the bandpass filter, the difference in area score for HER2 3 + and 2 + takes into account both the intensity of UCNP emission and the circular nature of HER2 expression as required by ASCO guidelines [41]. A similar area score of HER2 1 + and 0 was obtained. This can possibly be related to a minor amount of remaining UCNP clusters (non-specifically bound) present in the HER2 0 sample. Another explanation is the similarity of HER 1+, 0 expression making them hard to distinguish and quantify [10,42]. Improved washing steps to remove the UCNP clusters from the tissue slides and a better filtering procedure could help in optimizing the quantification at low HER2 expression levels. Though these minor clusters are present in all slides, they make more impact on results obtained for HER2 1 + and 0 as these slides have a minimal expression of HER2 receptors. However, our results in Fig. 4 show that we can measure the expression despite the presence of traces of UCNP clusters in the slides.

**Fig. 5.**
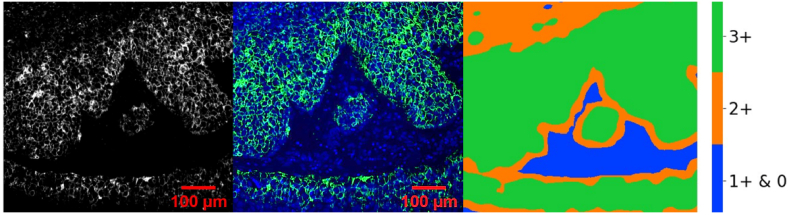
a) UCNP labeled HER2 3 + breast cancer tissue image taken at 976 nm excitation of UCNP and 800 nm emission b) overlay of UCNP (green) with DAPI (blue) emission. c) Area score assessment of a HER2 3 + breast cancer tissue labeled with UCNP.

To demonstrate the novel approach of the semi-quantified analysis of the HER2 expression in breast cancer tissue, a HER2 3 + breast cancer tissue slide was labeled with UCNPs as described in Fig. 1(b). The UCNP image of the slide is shown in Fig. 5(a). The cancer tissue was also counterstained with 4′,6-diamidino-2-phenylindole (DAPI), which stains the nuclei of cells. An overlap between the UCNP labeling and the counterstain is provided in Fig. 5(b). UCNP labeling along with DAPI staining helps to demarcate the HER2 overexpressed cells in relation to HER2 negative cells. The HER2 expression in UCNP images was semi-quantified by the Fourier method developed on the CPAs (Fig. 2). The pixel score image of breast cancer tissue was generated using the data in Fig. 5(a). The pixel scores are further color coded based on the HER2 expression thresholds estimated in Fig. 4. These HER2 expression levels are color coded and shown in Fig. 5(c). This provides a heat map of regions of different levels of HER2 expression. From this heat map it is evident that over 10% of cell area exhibits levels corresponding to HER2 3 + area scores. This is in line with the expected result for the imaged HER2 3 + breast cancer tissue. In addition, the signal-to-background value was calculated by taking the ratio of the peak counts in a UCNP labeled breast cancer image to the average of unspecific binding signal from a negative control breast cancer image acquired under the same experimental conditions. The signal-to-background value for the UCNP labeling was found to be 40.

In order to make a meaningful comparison, the breast cancer tissue was also labeled with classical DAB. The DAB precipitation was found in the cell membrane of HER2 positive breast cancer cells. The signal-to-background for DAB was calculated by taking the ratio of average background with no labeling to DAB labeled section of the breast cancer image. The signal-to-background for DAB was found to be 1.6, more details regarding the calculation can be found in the Supplement 1 S1. The current clinical practice for breast cancer treatment stratification relies on gold standard IHC using the HRP/DAB visualization system to provide information about the overexpression of HER2 and other biomarkers in breast cancer tissue [41]. DAB is renowned for high thermal and chemical stability making it an ideal choice for labeling in these aspects. However, in the DAB labeling, the signal is generated by the absorption of white light by the chromogen. The light attenuated by the DAB is fundamentally limited by the pathlength of white light in the tissue slide. Hence, at high DAB concentrations, no light gets through the slide. The only way is then to reduce the primary antibody concentration and redo the labeling but this influences the sensitivity on the lower end, thus the overall dynamic range is limited. This implies that image contrast provided by any absorption-based agent is relatively poor, requiring expert pathology experience and complicating the diagnosis and stratification processes. DAB imaging with white light further complicates the quantification process as it will require calibration of color, possibly drifting over time. On the contrary, the contrast in UCNP-labeling is obtained by the emission process which allows it to be limited only by the non-specific labeling of surrounding tissue. In this work, the contrast of the UCNP images with respect to background was found to be 40. This gives an enhancement factor of 25 for UCNP labeling as compared to DAB labeling. The signal-to-background, achieved in this work, can be further improved by optimizing the labeling process. It will be interesting to compare the performance of the proposed Fourier transform method with traditional HER2 level assessment by pathologists. One of the next steps in this work is to validate the proposed method with traditional method on a large dataset of breast cancer tissue.

In addition, breast cancer severity and treatment stratification are assessed based on the expression levels of multiple receptors like ER, PR, HER2, Ki-67. Simultaneous imaging of multiple biomarkers co-localized on a single tissue section can capture the spatial distributions and heterogeneity of biomarker expressions. This can allow not only for defining the tumour subtype, but also understanding the complex interplay of proteins and pathways involved in tumour formation and growth, all from a single slide image. In the context of breast cancer, for example, simultaneous imaging of key biomarker expressions, namely ER, PR and HER2, together with Ki67 and PD-L1 can shed light on the proliferative and immunogenic profile of different cells and regions in the tumour related to HER2- and ER-status, currently not available with standard IHC. Multiplexed biomarker profiling has the potential to create new paradigm in breast cancer understanding, thus impacting patients’ stratification and treatment outcomes [43]. Currently, it is difficult to co-localize DAB labeling together with another chromogen to simultaneously provide expressions of multiple receptors. In contrast, the narrow emission lines of UNCPs can be designed to easily multiplex biomarkers on the same tissue slide. Alternatively, fluorophores can be considered substitutes for DAB, however, the key challenges constitute autofluorescence background and photo-bleaching. Novel fluorophores like quantum dots can partially address photo-bleaching, but they still pose the challenge of auto-fluorescence background [44]. On the contrary, UCNP anti-Stokes’ shifted emission eliminates the challenge of Stokes-shifted auto-fluorescence. UCNPs are also known for their high photostability even at high excitation powers. Another aspect important to consider is the cost of optical system as UCNP-based labelling requires high power laser source at 976 nm. Though high power lasers are typically expensive, the lasers at 976 nm are affordable and widely available as they are used as optical pump in the fiber and solid-state lasers. This significantly brings down the cost of the microscope system.

In recent times, the increase in computation power has given rise to the development of novel AI/ML tools for pathological image analysis. The AI/ML tools are foreseen to create a new paradigm in histopathology to provide accurate predictions and aid pathologists in decision-making [8,16,45,46]. The performance of AI/ML models is enhanced when the contrast is high allowing models to effectively distinguish target receptors from the background. The above discussion highlights some of the key advantages of UCNPs in terms of multiplexing for multi-receptor labeling, high contrast for enhanced ML/AI training methods, zero auto-fluorescence background and high photo-stability as compared to other emerging fluorophores like quantum dots.

## 4. Conclusions

For the first time, we have reported the use of high contrast UCNP labeling to distinguish different levels of HER2 expression in HER2 CPA and HER2 positive breast cancer tissue. A simple Fourier transform algorithm trained on CPAs was sufficient to provide an objective semi-quantitative HER2 tissue assessment tool as demonstrated on a tissue sample. The UCNP breast cancer labeling was found to have a signal-to-background of 40 compared to the negative control, which is an enhancement of 25 times as compared to conventional DAB labeling. With an urgent need for better biomarker quantification and multiplexing methods for improved treatment stratification, the development of UCNP imaging and advancements in labeling processes shows great promise to be included in the clinical practice in the future as a reliable and accurate diagnostic tool.

## Data Availability

Data underlying the results presented in this paper are not publicly available at this time but may be obtained from the authors upon reasonable request.

## Supplemental document

See Supplement 1 for supporting content.
