# Supplementary material for: High contrast breast cancer biomarker semi-quantification and immunohistochemistry imaging using upconverting nanoparticles
Source: Biomed Opt Express. 2024 Jan 19;15(2):900–9. doi: 10.1364/BOE.504939 (PMC10890842; doi:10.1364/BOE.504939)
Supplement: Supplementary file 1 [file boe-15-2-900-s001.pdf]

Supplemental Document

Biomedical Optics EXPRESS

High contrast breast cancer biomarker semi-quantification and immunohistochemistry imaging using upconverting nanoparticles: supplement

SANATHANA KONUGOLU VENKATA SEKAR,[1,†,*] HUI MA,[1,2,†] KATARZYNA KOMOLIBUS,[1] GOKHAN DUMLUPINAR,[1,2] MATTHIAS J. MICKERT,[3] KRZYSZTOF KRAWCZYK,[3] AND STEFAN ANDERSSON-ENGELS[1,2]

[1] *Biophotonics@Tyndall, IPIC, Tyndall National Institute, Lee Maltings Complex, Dyke Parade, T12R5CP, Cork, Ireland*
[2] *Department of Physics, University College Cork, College Road, Cork, T12 K8AF, Ireland*
[3] *Lumito AB, Mårtenstorget 5, SE-223 51 Lund, Sweden*
[†] *These authors contributed equally to this work*
[*] *sanathana.konugolu@tyndall.ie*

This supplement published with Optica Publishing Group on 19 January 2024 by The Authors under the terms of the Creative Commons Attribution 4.0 License in the format provided by the authors and unedited. Further distribution of this work must maintain attribution to the author(s) and the published article's title, journal citation, and DOI.

Supplement DOI: https://doi.org/10.6084/m9.figshare.24972792

Parent Article DOI: https://doi.org/10.1364/BOE.504939

HIGH CONTRAST BREAST CANCER BIOMARKER SEMI-QUANTIFICATION AND IMMUNOHISTOCHEMISTRY IMAGING USING UPCONVERTING NANOPARTICLES

Supplementary Material (S1)

Formalin-fixed paraffin-embedded samples were sectioned in 4 µm thin slices on a microtome (Microm HM-360, Marshall Scientific, USA). The sections were placed on SuperFrost™ Plus microscope slides (HistoLab, Sweden). Dewaxing was performed by immersing the samples 2× for 5 min in Xylene followed by rehydration in 2× 5 min absolute ethanol, 5 min 96% ethanol, 5 min 70% ethanol, and 5 min in distilled H2O. For heat-induced antigen retrieval (HIER), the slides were transferred into a container with antigen-retrieval buffer (250 mL, EnVision FLEX high pH, Agilent, USA) that was pre-heated (95 °C) in a water bath. The HIER was conducted for 20 min at 95 °C. The container was removed from the water bath and allowed to cool down for 20 min.

The following steps were conducted at room temperature. Depending on the size of the sections 150–350 µL of the reagents were necessary to sufficiently cover the samples. The slides were transferred into a container with Tris-buffered saline containing 1 mM sodium fluoride (TBSF). Samples that were counterstained with hematoxylin were immersed in Mayer's Hematoxylin for 3 min followed by washing in TBSF (2× 1 min). Sections used for labelling with antibody-horseradish-peroxidase (HRP) conjugates were quenched with 0.3% H2O2 in TBSF for 10 min followed by a washing step in TBSF. All sections were encircled with a liquid blocker (ImmEdge Pen, Vector Laboratories, USA) and transferred to a Coplin jar with TBSF. The blocking buffer was applied to each section and incubated for 45 min followed by washing in TBSF for 1 min.

UCNP Labelling

For UCNP labellings, endogenous biotin was blocked using a ready-to-use streptavidin solution (20 min, SP-2002, Vector Laboratories) followed by a biotin solution (20 min, SP-2002, Vector Laboratories). Non-specific binding sites were blocked using TBSF containing 10% SuperBlock (37535, ThermoFisher Scientific) The slides were washed 2× for 1 min in TBSF after each blocking step.

The primary anti-HER2 antibody (A0485, Agilent) was diluted to 1 µg/mL in dilution buffer (TBSF + 0.05% Tween20, 10% SuperBlock) added to the sections, and incubated for 60 min. Negative controls were incubated with dilution buffer without the primary antibody. The slides were washed 2× 1 min with TBSF.

Slides for traditional IHC labelling (HRP/DAB) were incubated with a ready-to-use anti-rabbit secondary antibody-HRP conjugate (ImmPress® HRP Goat Anti-Rabbit IgG Polymer Detection Kit Peroxidase, Vector Laboratories) for 45 min and washed 3× 1 min with TBSF. For the DAB reaction, one droplet of the DAB substrate per milliliter substrate buffer (EnVision Flex Mini Kit, High pH, Agilent) was prepared. The antibody-HRP labelled slides were incubated for 10 min with substrate dilution and washed 3×1 min with TBSF. The DAB slides were then dehydrated by immersing them subsequently for 3× 1 min in 70% ethanol, 96% ethanol, and absolute ethanol, and at the end in Xylene for 5 min. Mounting of the coverslip was performed with Pertex as a mounting medium.

Slides for UCNP labelling were incubated with 2 µg/mL biotinylated anti-rabbit secondary antibody in dilution buffer (711-005-152, Jackson Immunoresearch, USA) for 60 min. The slides were washed 2× 1 min with TBSF and incubated for 45 min with a UCNP-streptavidin conjugate (SCIZYS reagent, Lumito, Sweden) in dilution buffer. After 3× washing for 1 min in TBSF, the slides were mounted with an aqueous mounting medium (Fluoroshield™, Sigma

Aldrich). Slides counterstained with DAPI were instead mounted with a DAPI-containing mounting medium (ab104139, Abcam, UK).

A Köhler reflected light illumination and imaging microscope system was employed to image the UCNPs labelled samples. A collimated 976nm laser (dst11-t193-h2o, Ostech) beam passed through two identical microlens arrays (10 x 10mm, 500μm Pitch, 1.2° Divergence, Edmund Optics), and then was magnified by a telescope system (LB1779-B, LB1676-B, Thorlabs). After that, the beam was focused by the objective lens (20X Nikon CFI60 TU Plan Epi ELWD, Nikon) of the microscope to form a 725x725 μm spot to illuminate the sample. The anti-Stokes emission from UCNPs was filtered by a 950 nm short-pass filter (FF01-950/SP-25, Semrock) to block the reflected laser light.

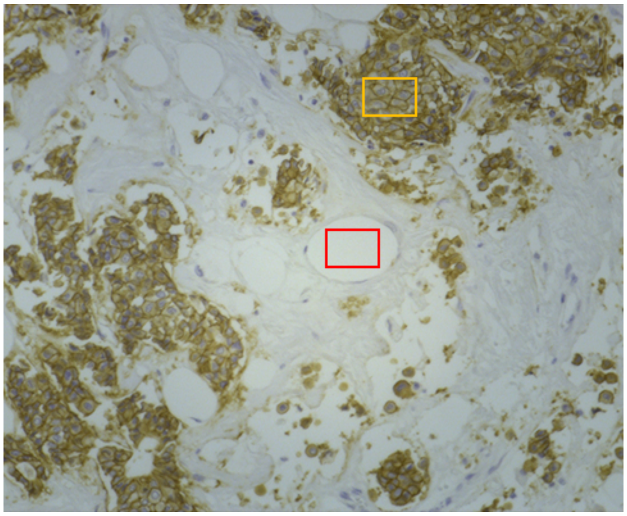

Fig. S1. DAB labeled HER2 3+ breast cancer image with DAB signal (yellow box) and background area indicated with a red box.

The signal-to-background for DAB was calculated by taking the ratio of average background with no labeling (red box in Sup.Fig.1) to DAB (yellow box in Fig. S1.) labeled section of breast cancer image. To perform the calculation, the signal was averaged across RGB pixels and the signal-to-background for DAB was found to be 1.6.
